# T-Cadherin and the Ratio of Its Ligands as Predictors of Carotid Atherosclerosis: A Pilot Study

**DOI:** 10.3390/biomedicines9101398

**Published:** 2021-10-05

**Authors:** Alexander Balatskiy, Marina Teterina, Alexandra Pisaryuk, Irina Balabanenko, Alexey Kadrev, Anastasia Tishuk, Maria Balatskaya, Larisa Samokhodskaya, Sergey Boytsov, Natalia Kalinina, Vsevolod Tkachuk

**Affiliations:** 1 Medical Scientific and Educational Centre, Lomonosov Moscow State University, 119192 Moscow, Russia; IBalabanenko@mc.msu.ru (I.B.); akadrev@yandex.ru (A.K.); nastenka702@mail.ru (A.T.); slm@fbm.msu.ru (L.S.); tkachuk@fbm.msu.ru (V.T.); 2 Federal Center of Brain Research and Neurotechnologies, 117513 Moscow, Russia; 3 Department of Internal Medicine, Peoples Friendship University of Russia (RUDN), 117292 Moscow, Russia; teterina-ma@rudn.ru (M.T.); pisaryuk-as@rudn.ru (A.P.); 4 Russian Medical Academy of Continuous Professional Education, 125445 Moscow, Russia; 5 Faculty of Medicine, Lomonosov Moscow State University, 119991 Moscow, Russia; m.balatskaya@gmail.com (M.B.); n_i_kalinina@mail.ru (N.K.); 6 National Medical Research Center of Cardiology, 121552 Moscow, Russia; prof.boytsov@gmail.com

**Keywords:** atherosclerosis, T-cadherin, high molecular weight adiponectin, intima-media thickness

## Abstract

In the cardiovascular system, atherogenic low-density lipoproteins (LDL) and the protective hormone adiponectin bind to the same receptor, T-cadherin. In this study, we tested the hypothesis that the ratio of circulating LDL to high-molecular weight (HMW) adiponectin could predict the development of atherosclerosis. Using enzyme-linked immunosorbent assay, we measured the level of circulating HMW adiponectin in the blood of donors together with ultrasound measuring of intima-media thickness (IMT) of carotid arteries. Single-nucleotide polymorphisms in the T-cadherin gene were identified using polymerase chain reaction. We found that carotid artery IMT is inversely correlated with the level of HMW in male subjects. We also found that the G allele of rs12444338 SNP in the T-cadherin gene correlates with a lower level of circulating T-cadherin and thinner IMT and therefore could be considered as an atheroprotective genotype. Despite our data, we could not provide direct evidence for the initial study hypothesis. However, we did uncover an important correlation between circulating T-cadherin and thinner carotid IMT.

## 1. Introduction

The causes of the atherosclerotic lesions’ formation and methods for the prevention of atherosclerosis have been studied for more than 100 years. It was demonstrated that the main mechanism of atherogenesis is the accumulation of low-density lipoproteins (LDL) in the vessel wall with the subsequent development of inflammation when increasing the quantity of extracellular matrix in the affected area and the formation of a plaque. LDL influence the vascular cells not only due to the transport of cholesterol into the cells [1] but also due to their ability to induce intracellular signaling (which is necessary for the normal functioning of the cardiovascular system) [2]. LDL in smooth muscle (SMC), endothelial cells, and fibroblasts induce fast (seconds, minutes) hormone-like intracellular signaling: phosphoinositide turnover, increasing the concentration of intracellular calcium ions, and the activation of protein kinase C. This signaling leads to cell proliferation and migration [2,3,4,5,6,7,8,9,10,11,12]. LDL not only triggers the calcium signaling but also enhances the effect of calcium-mobilizing substances: angiotensin II, epinephrine, thrombin, fibroblast growth factor, platelet-derived growth factor, and both insulin-like and epidermal growth factors [6,7,10,11,13,14,15,16,17]. These effects of LDL, as with its accumulation within cells, are also pro-atherogenic.

Our research group identified glycosylphosphatidylinositol(GPI)-anchored T-cadherin protein as a receptor mediating the “hormone-like” effects of LDL [18]. Such intracellular signaling from LDL is induced in vitro at concentrations ten times lower than physiological signaling. This, for a long time, led to the question: is this signaling permanent as a result of the constant occupation of the T-cadherin by LDL?

In 2004, adiponectin, another ligand of T-cadherin with higher affinity, was discovered [19]. Unlike LDL, adiponectin has an anti-atherogenic effect. The concentration of this hormone of adipose tissue decreases in obesity [20]. In early studies, it was found that its concentration also significantly decreases in various cardiovascular diseases, metabolic syndromes (low adiponectin levels are associated with obesity and insulin resistance), and type 2 diabetes [20,21,22,23,24,25,26,27,28]. Moreover, various anti-atherogenic effects of adiponectin on macrophages, smooth muscle and endothelial cells, leukocytes, and platelets have been demonstrated in experiments (see [29] for review). It affects the migration of lymphocytes to atherosclerotic lesions, reduces the level of C-reactive protein, and inhibits TNF-α and NF-κB-mediated pro-inflammatory intracellular signaling [30]. In human studies, it was shown that low adiponectin levels are associated with an increased carotid intima-media thickness (IMT) (a sign of the initial stages of atherosclerosis) [31]. Even though these data are not reproduced in all studies, meta-analysis suggests that a low level of adiponectin is associated with a greater carotid IMT [32]. The most physiologically active circulating form of adiponectin is the high molecular weight (HMW) [33]. The inverse correlations of plasma concentrations with body mass index, cardiovascular diseases, insulin sensitivity, and diabetes mellitus were shown exactly for this form [20,33,34,35,36,37,38,39]. In vitro data suggests that high molecular weight adiponectin causes phosphorylation of adenosine monophosphate-dependent protein kinase (AMPK) and prevents apoptosis of endothelial [37].

It has been proven that T-cadherin, but not the AdipoR1 and AdipoR2 receptors, binds a significant portion of blood adiponectin on the cells’ surfaces in the cardiovascular system and mediates the action of this hormone on them [40,41,42]. In T-cadherin gene knockout mice, adiponectin does not bind to tissues, and its concentration in the blood increases fivefold [40,41,42]. The association between adiponectin and T-cadherin concentrations is also shown in humans; a number of single nucleotide polymorphisms (SNPs) in the T-cadherin gene affecting its expression level correlate with plasma adiponectin concentration [43,44,45,46,47,48,49,50] and cardiovascular diseases [43,47,51,52,53].

In Ranscht’s laboratory, it was shown that the knockout of the T-cadherin gene in mice leads to the abolition of the protective effect of adiponectin, which is manifested in the development of excessive cardiac hypertrophy and an increase in the myocardial infarction area [40]. Using the same knockout animal model, it was demonstrated that T-cadherin is essential for adiponectin-mediated revascularization [42]. Finally, in 2017, it was shown that the adiponectin-T-cadherin complex protects against neointima proliferation and the formation of atherosclerotic plaques [41]. Thus, it was demonstrated in the mouse model that, in the absence of T-cadherin, the concentration of adiponectin in the blood is high. At the same time, however, atherosclerotic lesions develop. It can be assumed that in cases of the “adiponectin paradox” (high mortality associated with a high concentration of adiponectin in the blood (data summarized in [54]), adiponectin does not have a protective effect, since its concentration in tissues is low, despite the high content in the blood plasma. We suggest that such a decrease in the concentration of adiponectin in tissues may occur as a result of the competitive displacement of adiponectin from the T-cadherin receptor by LDL in cases of dyslipidemia. The coefficients of dissociation were measured by different methods for LDL [12,55,56] and for adiponectin [19,57]. Previously, we supposed a possible competition between them [58,59].

The normal plasma concentration of adiponectin is 2–20 µg/mL, which is several orders of magnitude higher than the concentration of many cytokines and hormones [20]. A significant part of adiponectin is normally associated with T-cadherin in tissues [40,41,42,58,59,60].

The concentration of adiponectin in the blood, unlike other adipose-tissue-originating hormones, decreases with obesity (while the LDL level increases). Obesity and metabolic syndrome, known as pro-atherosclerotic conditions, lead to a significant change in the ratio of adiponectin to LDL in the blood, presumably resulting in increasing the probability of LDL binding to T-cadherin, which can trigger adverse effects. According to our new hypothesis about the development of atherosclerosis, for the onset of atherogenesis, it is not the absolute values of LDL or adiponectin in the blood are important but rather the ratio between them.

To test this hypothesis, we checked if the ratio between LDL (or apolipoprotein B (ApoB)) and HMW adiponectin concentrations are associated with the initial stages of atherosclerosis in humans. The measurement of carotid intima-media thickness (IMT) was used to evaluate the presence of early-stage atherosclerosis. Since the concentration of T-cadherin and adiponectin in advanced atherosclerotic lesions is high [41,61] and may distort the HMW adiponectin levels in plasma, all patients with developed atherosclerotic plaques were excluded from the study.

Adiponectin and T-cadherin levels are at least partly genetically determined. That is why we checked not only the influence of the LDL to HMW adiponectin ratio on the IMT but also the role of single nucleotide polymorphisms (SNPs) in the CDH13 gene (encoding T-cadherin) in early atherosclerosis formation. Since some genome-wide association studies demonstrated the link between SNPs in the CDH13 gene and arterial hypertension (AH) [49,53], we also compared patients with AH and with normal blood pressure.

## 2. Materials and Methods

### 2.1. Patients and Eligibility Criteria

A cross-sectional observational multicenter cohort study was approved by the Local Ethics Committee of the Medical Scientific and Educational Center, Lomonosov Moscow State University (protocol No. 2/20 dated 16 March 2020). The study was conducted in accordance with the guidelines of the Declaration of Helsinki. All participants gave written informed consent to take part in the study. Male and female patients >30 years old with moderate cardiovascular risk (SCORE 1–4%) were enrolled in the study. Patient information was obtained from medical charts including age, sex, smoking, history of cardiovascular diseases, and therapy. Physical examination with a measurement of blood pressure, heart rate, height, and weight were performed.

In our study, only patients who do not receive statins (to prevent impact on LDL levels) and do not have atherosclerotic plaques (to prevent impact on HMW adiponectin and T-cadherin levels) according to anamnesis and ultrasound data were included. The following exclusion criteria were also applied:

- Total cholesterol level >8 mmol/L (>310 mg/dL) or blood pressure >180/110 mm Hg
- Diabetes
- Established familial hypercholesterolemia
- The level of triglycerides is more than 5.7 mmol/L
- Established systemic inflammatory diseases
- Malignant neoplasm, or another disease (not related to the heart), limiting life expectancy to <three years
- Current abuse of alcohol and/or psychoactive drugs
- Established liver failure (total bilirubin >3 mg/dl) or a history of cirrhosis with signs of portal hypertension
- Pregnancy, lactation

### 2.2. Ultrasonography

All patients underwent Duplex Doppler ultrasonography of the brachiocephalic arteries and arteries of the lower extremities (Epiq 5, Philips Medical Systems B.V, Netherlands, ultrasound system with linear transducers 12-3 MHz and 18-5 MHz) by qualified researchers. In duplex scanning of the brachiocephalic branches of the aortic arch on the left and right, the following were investigated: the distal part of the brachiocephalic trunk, the proximal (before the divergence of the vertebral arteries) segments of the subclavian arteries, the common carotid arteries throughout, the external carotid arteries in the proximal segments, the internal carotid arteries in the extracranial parts, and the vertebral arteries (segments V1 and V2). During duplex scanning of the arteries of both lower extremities, the following were examined: common femoral, deep and superficial femoral arteries, popliteal artery, lower leg arteries (anterior and posterior tibial arteries, peroneal artery), arteries of the dorsum of the foot. During duplex scanning, the IMT was measured at a typical measurement site in the common carotid artery.

The detection of atherosclerotic plaque in any of the examined arteries was the criterion for excluding the patient from the study.

### 2.3. Laboratory Tests

All patients underwent laboratory tests. A blood count was performed using a Sysmex XN-1000 automated analyzer, and glycated hemoglobin levels were measured using a DCA Vantage analyzer. All biochemical blood tests, except for the analysis of HMW adiponectin and T-cadherin, were performed using a Beckman Coulter AU480 biochemical analyzer. HMW adiponectin was measured in serum using Human HMW Adiponectin/Acrp30 Quantikine ELISA Kit (DHWAD0, R&D Systems, Minneapolis, MN, USA). T-cadherin was measured in serum using Human Cadherin 13 ELISA Kit (RAB1029, Sigma-Aldrich, Burlington, MA, USA). DNA was isolated from whole venous blood using QIAamp DNA Blood Mini Kit (Qiagen, Venlo, The Netherlands) and QIACube automated station (Qiagen) according to manufacturer’s protocols. CDH13 single nucleotide polymorphisms (SNPs) genotyping was performed using corresponding TaqMan SNP Genotyping Assays (Applied Biosystems, Waltham, MA, USA) and 7500Fast real-time PCR machine (Applied Biosystems) according to manufacturer’s protocols.

### 2.4. Statistical Analysis

Continuous variables were expressed as median (interquartile range) and analyzed with the Mann–Whitney U test. For multiple comparisons, the Kruskal–Wallis test was used. The Spearman rank correlation coefficient test was used for the correlation of continuous variables. For binary variables, Yates corrected χ^2^ was used. SNP data were analyzed using the SNPStats online tool (version 0.96, Sole et. al., Barcelona, Spain) [62]. This tool uses logistic regression models and calculates the OR (odds ratio) for each genotype with respect to the reference genotype. According to the number of minor alleles needed in order to modify the risk, five inheritance models are estimated: codominant (every genotype gives a different and non-additive risk), dominant (a single copy of minor allele is enough to modify the risk), recessive (two copies of the minor allele are necessary to change the risk), overdominant (heterozygous are compared to a pool of both allele homozygous), and log-additive (each copy of the minor allele modifies the risk in an additive form). Akaike information criteria (AIC) was used to choose the inheritance model that best fits the data (lower AIC values indicate better models). In all figures, squares show medians; whiskers show upper and lower quartiles.

## 3. Results

### 3.1. Baseline Characteristics of the Subjects

Forty-two patients were enrolled in the study; seven of them were excluded after ultrasonography due to the presence of atherosclerotic plaques. The main characteristics of the 35 included patients are shown in Table 1.

### 3.2. Correlations between Laboratory Data and Carotid IMT

We tested correlations between parameters of interest (LDL, ApoB, HMW adiponectin, and T-cadherin levels, HMW adiponectin/LDL and HMW adiponectin/ApoB molar ratios) and carotid IMT and did not find significant correlations. All correlation coefficients are present in Table A1 (Appendix A). Despite age is considered as a cardiovascular risk factor, we did not find any significant correlations between age and IMT or laboratory data.

Since adiponectin levels are dependent on sex, we divided subjects into male and female subgroups. HMW adiponectin was higher in females (15.45 (11.71; 22.62) nM vs. 10.68 (7.81; 13.05) nM, *p* = 0.014), other differences in laboratory data were non-significant. It is known that adiponectin and particularly HMW adiponectin levels are lower in obesity; we also analyzed correlations in obese and non-obese patients (body mass index (BMI) ≥30 and <30 accordingly). In our dataset, there were no differences in laboratory data between obese and non-obese patients.

In male subjects, we found correlations between carotid IMT and HMW adiponectin. Some correlations between carotid IMT, HMW adiponectin/LDL, and HMW adiponectin/ApoB molar ratios were also present (Table 2).

In female patients, no correlations between carotid IMT and laboratory data were found, but plasma T-cadherin levels were significantly (*p* < 0.05) correlated with HMW adiponectin levels (ρ = 0.397). The same correlation was found for the HMW adiponectin/ApoB molar ratio (ρ = 0.396).

No significant correlations between carotid IMT and laboratory data were demonstrated in subgroups divided by BMI (obese and non-obese patients).

**Figure 1:**
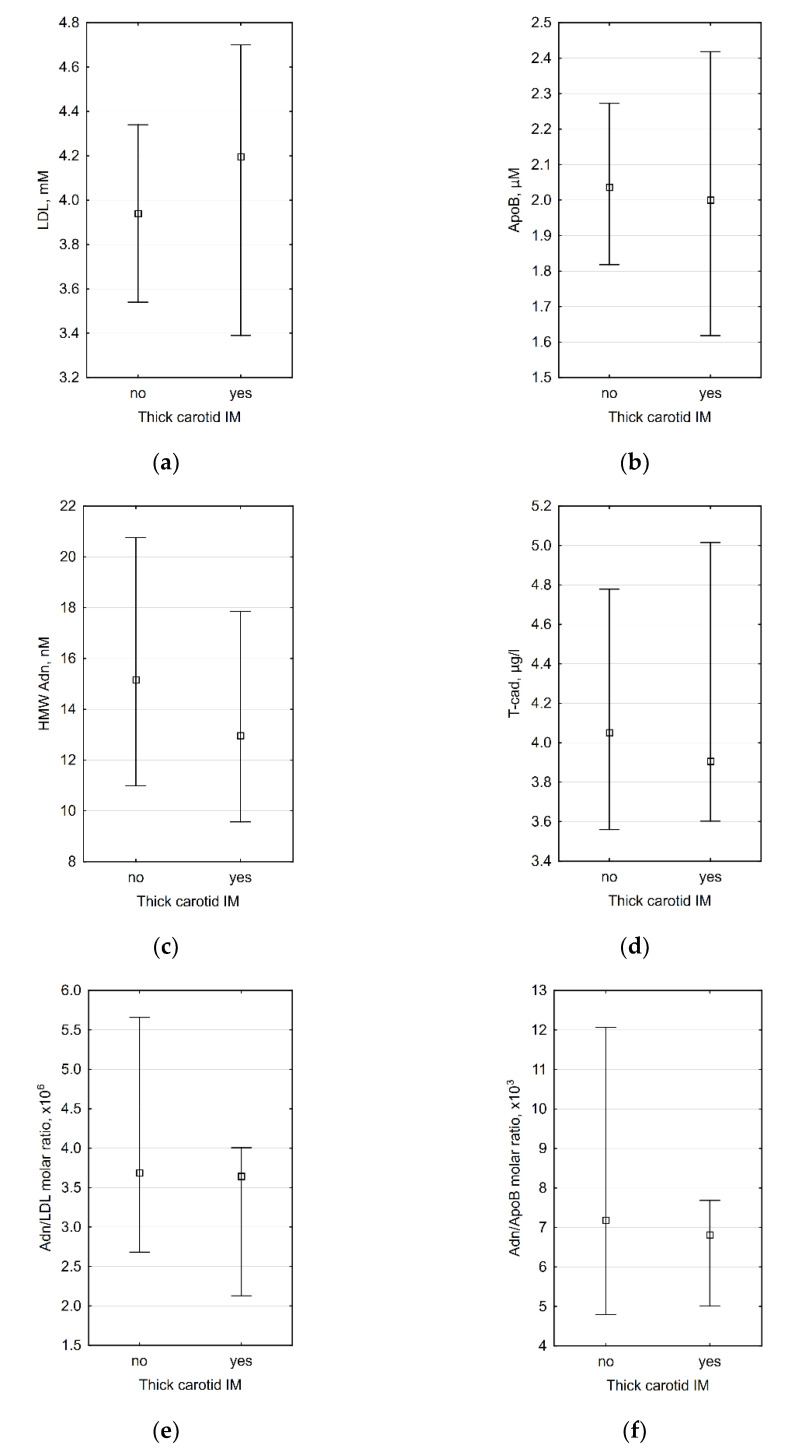
Association between presence of thick (>0.9 mm) carotid intima-media complex and (**a**) plasma LDL level (*p* = 0.661), (**b**) plasma ApoB level (*p* = 0.711), (**c**) plasma HMW adiponectin level (*p* = 0.469), (**d**) plasma T-cadherin level (0.853), (**e**) HMW adiponectin/LDL molar ratio (0.578), and (**f**) HMW adiponectin/ApoB molar ratio (*p* = 0.625). *n* = 35 for all diagrams.

Since carotid IMT >0.9 mm is considered abnormal by the European Society of Cardiology [63,64], we also divided all patients into subgroups with thick (>0.9 mm) and thin (≤0.9 mm) carotid intima-media. The comparison of laboratory data between these subgroups did not reveal any significant differences (Figure 1).

### 3.3. Association between Laboratory Data and the Presence of Arterial Hypertension (AH)

**Figure 2:**
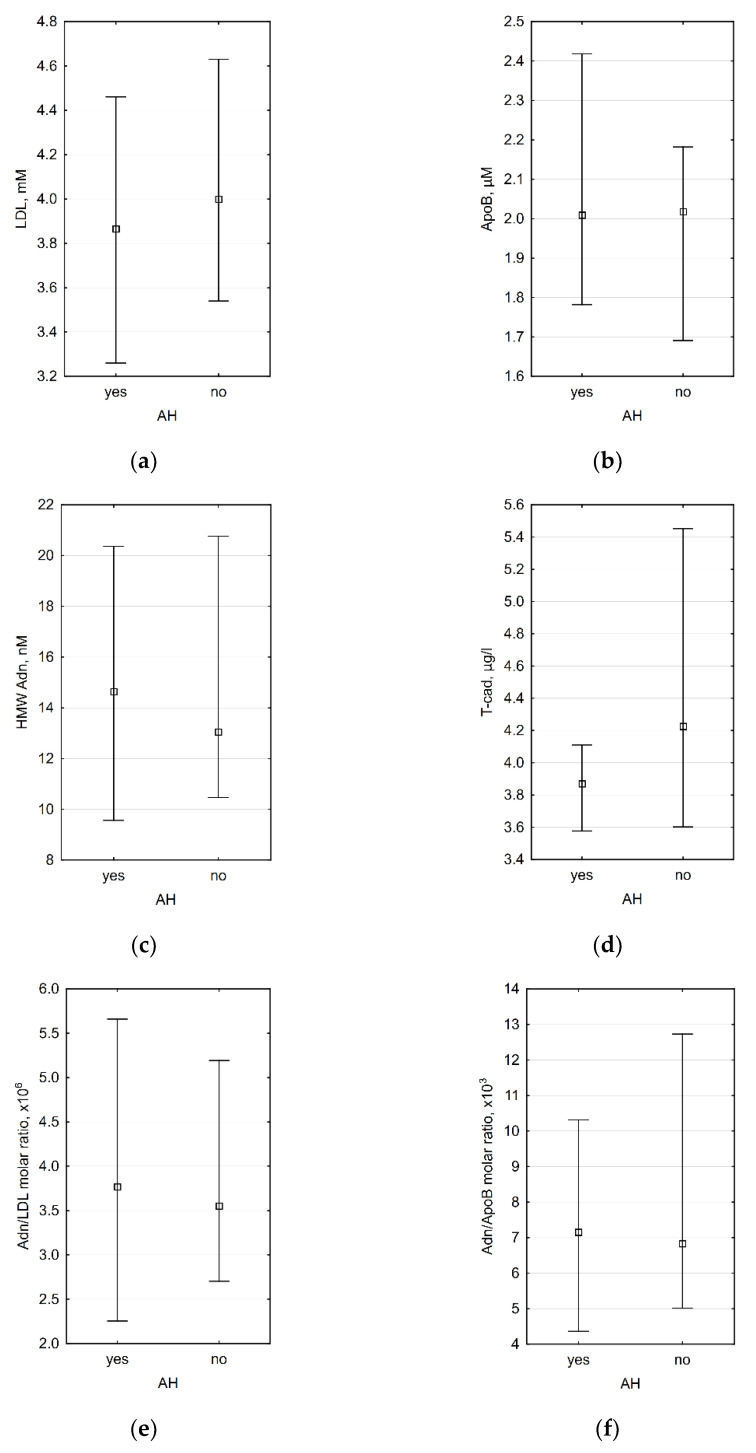
Association between presence of AH and (**a**) plasma LDL level (*p* = 0.609), (**b**) plasma ApoB level (*p* = 0.668), (**c**) plasma HMW adiponectin level (*p* = 0.779), (**d**) plasma T-cadherin level (0.181), (**e**) HMW adiponectin/LDL molar ratio (0.961), and (**f**) HMW adiponectin/ApoB molar ratio (*p* = 0.779). *n* = 35 for all diagrams.

According to genome-wide association studies, genetically determined T-cadherin and adiponectin levels may correlate with blood pressure and the presence of AH [49,53]. We did not discover any significant differences between patients with AH and those with normal blood pressure (Figure 2).

### 3.4. CDH13 SNP Data

We studied three SNPs in CDH13 (T-cadherin) gene: rs4783244, rs12051272, and rs12444338. According to the literature data, all these SNPs are linked to adiponectin levels [43,46,48,65,66,67], and rs12444338 is also linked to T-cadherin levels [46].

**Figure 3:**
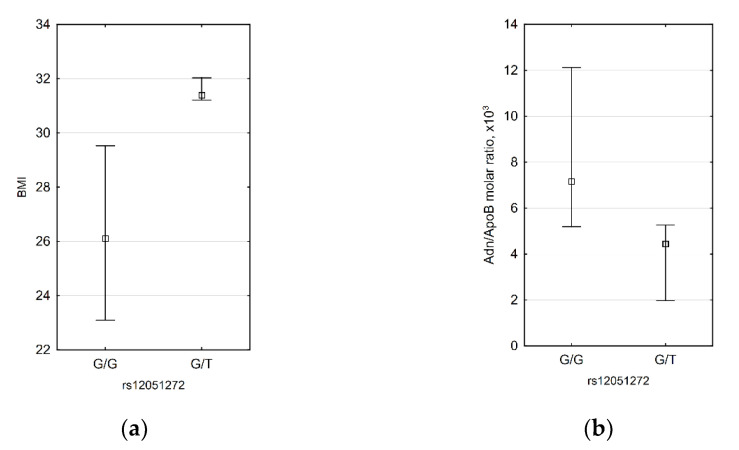
Significant differences between rs12051272 T allele carriers and non-carriers: subjects with G/T genotype have higher BMI (**a**) and lower HMW adiponectin/ApoB molar ratio (**b**). *n* = 35 for both diagrams.

For rs12051272, we demonstrated that subjects with G/T genotype have higher BMI (31.4 (31.2; 32.0) vs. 26.1 (23.1; 29.5), *p* = 0.0311) and lower HMW adiponectin/ApoB molar ratio (4.44 × 10^3^ (1.97 × 10^3^; 5.26 × 10^3^) vs. 7.15 × 10^3^ (5.19 × 10^3^; 12.12 × 10^3^) (Figure 3). Inverse correlations between BMI and HMW adiponectin/ApoB molar ratio were demonstrated by us for men, but since in male patients there was only one subject with G/T genotype, it is not possible to say if rs12051272 has an effect only in men.

**Figure 4:**
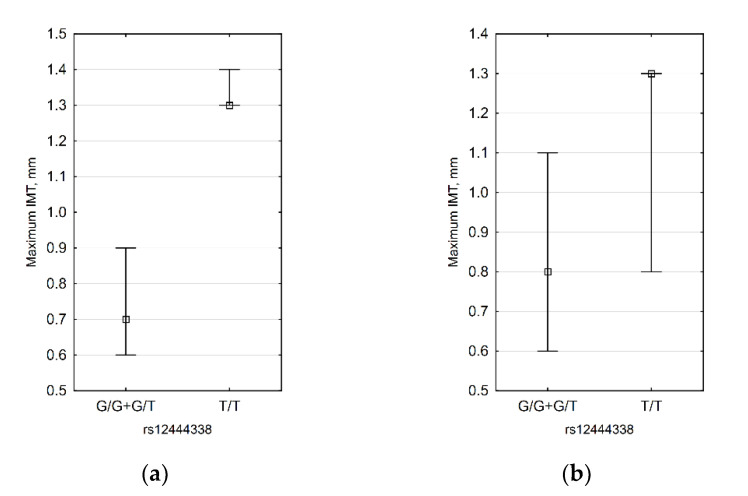
Significant differences in IMT between rs12444338 G allele carriers and non-carriers: (**a**) obese patients (*n* = 10, *p* = 0.020) and (**b**) females (*n* = 25, *p* = 0.047).

For rs12444338, we demonstrated the association of the G allele with thin IMT (Table 3). When IMT values were compared between G allele carriers and non-carriers, we found that the association is present in obese and female patients (Figure 4). The subgroup of obese female patients contained only nine subjects, so only a difference in the right carotid IMT was observed (0.6 mm vs. 1.3 mm).

**Figure 5:**
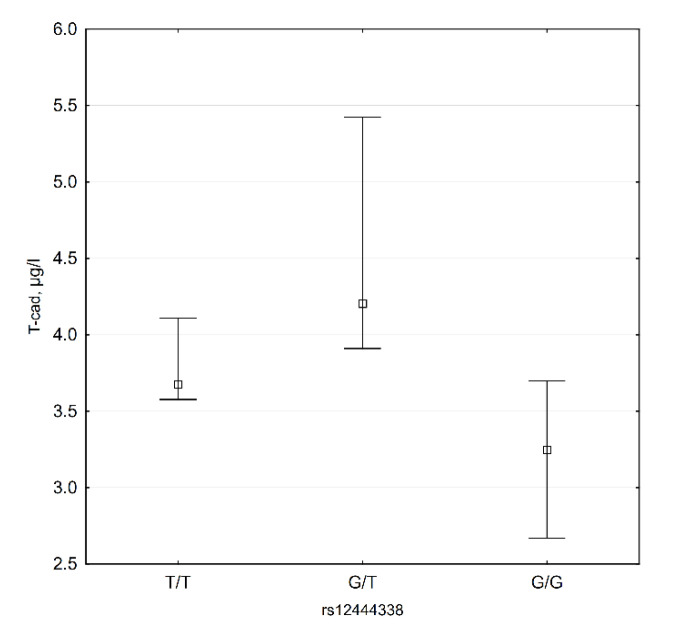
CDH13 rs12444338 genotypes and T-cadherin plasma levels (*n* = 35).

Our data show that plasma T-cadherin levels are lower in G/G subjects (Figure 5). Median T-cadherin plasma level in these subjects is 3.25 (2.67; 3.70) µg/L, while in other genotypes this level is 4.061 (3.62; 5.07) µg/L (*p* = 0.018).

Additionally, we demonstrated that rs12444338 G allele carriers have higher ApoB and LDL levels comparing to T/T genotype carriers—2.15 (1.98; 2.42) µM vs. 1.82 (1.62; 2.02) µM (*p* = 0.023) and 4.33 (3.62; 4.70) mM vs. 3.78 (3.12; 4.04) mM (*p* = 0.017) accordingly.

We showed no significant correlations for rs4783244.

## 4. Discussion

This study allowed us to test the hypothesis that the ratio of HMW adiponectin plasma level to LDL plasma level is a better predictor for early atherosclerosis than LDL or HMW adiponectin levels alone. Carotid IMT was chosen as a marker of early atherosclerosis. Our research has some limitations because data concern only early atherosclerotic changes. Nevertheless, we believe that biochemical markers of early atherosclerosis are very important from the clinical point of view. Early discovery of atherosclerotic lesions by biochemical screening methods may prevent the development of atherosclerosis complications.

The significance of LDL for atherosclerosis development is well established and demonstrated in many studies. Many anti-atherogenic effects were demonstrated in vitro for various cells (data reviewed in [29]). Human studies are also demonstrating an influence of low adiponectin levels on IMT (data reviewed in [32]). HMW adiponectin is considered as most active, and its levels inversely correlate with different pro-atherogenic conditions and cardiovascular diseases [20,33,34,35,36,37,38,39]. Nevertheless, a few data about the link between HMW adiponectin and carotid IMT are available. Several studies demonstrated no correlations between HMW adiponectin and carotid IMT in type 2 diabetic patients [68], obstructive sleep apnea patients [69], obese/overweight children [70], and in hemodialysis patients [71]. Some authors showed correlations between HMW adiponectin and carotid [72] or coronary [73] atherosclerotic plaques, but not IMT. In obese adolescents, the strong correlation between HMW adiponectin and carotid IMT was demonstrated, and after multiple testing, the HMW subfraction showed a better correlation to IMT compared with total adiponectin [74]. As far we know, there are no studies investigating the effects of HMW adiponectin on carotid IMT in persons with low or moderate cardiovascular risk. Our data demonstrate the link between HMW adiponectin and carotid IMT in both obese and non-obese male patients. It is known that total adiponectin levels are lower in men than in women [20], and the percentage of HMW adiponectin is lower in men, too [33]. This is consistent with our data demonstrating lower HMW adiponectin in men. In our study, we did not find the effect of HMW adiponectin on IMT in women.

In this paper, we studied the effects of the ratio of T-cadherin ligands for the first time. Previously it was only shown that HMW to total adiponectin ratio is related to the stable state of IMT in hemodialysis patients [71]. Takamura with colleagues also demonstrated that leptin to high-molecular-weight adiponectin ratio is independently correlated with carotid intima-media thickness in men but not in women [75]. We found some correlations between carotid IMT, HMW adiponectin/LDL, and HMW adiponectin/ApoB molar ratios, but they were obviously attributed to HMW adiponectin only (see Table 3).

Data from genetic studies demonstrate a possible link between SNPs in the CDH13 gene and blood pressure. Thus, we checked if levels T-cadherin and their ligands are correlated with AH, but no correlations were found.

It is also known that some SNPs in the CDH13 gene are associated with plasma adiponectin levels. For rs12051272, the T allele is associated with lower adiponectin levels. We discovered the correlation between the presence of this allele and the lower HMW adiponectin/ApoB molar ratio. T-allele was also associated with higher BMI. It is well known that adiponectin level is lower in obese patients [20]. Our data raise the question about the causal relationship between body mass and adiponectin level that is genetically determined, at least partly. We also can suppose that the ratio of T-cadherin ligands better correlates with genetically altered T-cadherin levels than levels of its ligands alone.

For rs12444338, we demonstrated that G allele carriers have lower IMT. Our data do not support previous research by Lee et al., who demonstrated lower IMT in GG vs. TT genotype carriers [47]. This inconsistency may be due to different populations studied and low sample sizes in both studies. In our case, maximal IMT measurements were used, while Lee et al. used mean IMT. We believe that maximal IMT is the better marker for early atherosclerosis, but our dataset showed similar results for maximal and mean IMT (data not shown). We also demonstrate lower plasma levels of T-cadherin in G/G subjects. It was unexpected after the demonstration of a 2.2-times increase of CDH13 promoter activity for the G allele by luciferase assay [46]. Higher T-cadherin expression may does not mean its higher plasma level, and this question needs further research. However, it needs to be noted that we showed higher ApoB and LDL levels in G allele carriers. It is possible to speculate that the G allele is linked to a lower tissue level of the receptor (T-cadherin), and this may lead to a higher level of the ligand (LDL) in plasma.

The correlation between rs12444338 G allele and lower IMT was confirmed in obese patients and females. Female sex and obesity have opposite effects on levels of T-cadherin ligands and, as far we know, have no effect on the level of T-cadherin itself. Females and persons with normal BMI have lower LDL and higher adiponectin levels comparing to males and obese persons accordingly [20,76]. Further investigations are needed to clarify the relationship between SNP in the T-cadherin gene, levels of its ligands, sex, BMI, and IMT.

## 5. Conclusions

This is the first study of the T-cadherin ligands (HMW adiponectin and LDL) ratio and its effect on early atherosclerosis. Despite the initial study hypothesis was not proven directly, we demonstrate a possible link between T-cadherin, its ligands levels, and carotid IMT. The inverse correlation between carotid artery IMT and HMW adiponectin levels in male subjects with moderate cardiovascular risk was demonstrated. We discovered an unexpected link between the G allele of rs12444338 SNP in the T-cadherin gene and two parameters, the lower level of circulating T-cadherin and the thinner intima-media.

## Figures and Tables

**Table 1:** Basic data of individuals included in the study.

	IMT ≤ 0.9 mm, *n* = 21	IMT > 0.9 mm, *n* = 14	p IMT ≤ 0.9 vs. IMT > 0.9 mm	AH, *n* = 18	No AH, *n* = 17	p AH vs. No AH
Sex Male, *n* (%) Female, *n* (%)						
	6 (28.6) 15 (71.4)	4 (28.6) 10 (71.40)	0.703	5 (27. 8) 13 (72.2)	5 (29.4) 12 (70.6)	0.789
Age, years	54 (50; 59)	52 (48; 60)	0.661	51.5 (48; 60)	55 (50; 59)	0.437
Obesity, *n* (%)	6 (28.6)	4 (28.6)	0.990	7 (38.9)	3 (17.7)	0.310
AH, *n* (%)	12 (57.1)	6 (42.9)	0.629	18 (100)	0 (0)	-
IMT >0.9 mm, *n* (%)	0 (0)	14 (100)	-	6 (33.3)	8 (47.1)	0.629

**Table 2:** Significant Spearman correlation coefficients between carotid IMT and laboratory data in male patients.

	ApoB	HMW Adn	Adn/ApoB Molar Ratio	Adn/LDL Molar Ratio
Carotid IMT, right	0.308	−0.828 *	−0.656 *	−0.779 *
Carotid IMT, left	0.080	−0.722 *	−0.489	−0.538
Carotid IMT, max.	0.095	−0.746 *	−0.502	−0.575
BMI	0.693 *	−0.612	−0.721 *	−0.612

* Significant correlations at *p* < 0.05.

**Table 3:** Association between rs12444338 and the presence of thick intima-media.

Model	Genotype	IMT ≤ 0.9 mm. *n*(%)	IMT > 0.9 mm. *n*(%)	OR (95% CI)	*p*	AIC
Codominant	T/T	5 (23.8%)	8 (57.1%)	1.00	0.032	46.2
	G/T	12 (57.1%)	6 (42.9%)	0.31 (0.07–1.38)		
	G/G	4 (19.1%)	0 (0%)	0.00 (0.00–NA)		
Dominant	T/T	5 (23.8%)	8 (57.1%)	1.00	0.045	47.1
	G/T-G/G	16 (76.2%)	6 (42.9%)	0.23 (0.05–1.01)		
Recessive	T/T-G/T	17 (81%)	14 (100%)	1.00	0.035	46.7
	G/G	4 (19.1%)	0 (0%)	0.00 (0.00–NA)		
Overdominant	T/T-G/G	9 (42.9%)	8 (57.1%)	1.00	0.41	50.4
	G/T	12 (57.1%)	6 (42.9%)	0.56 (0.14–2.21)		
Log-additive	---	---	---	**0.23 (0.06–0.84)**	**0.015**	**45.1**

OR, odds ratio; (95%) CI, 95% confidence interval. Significant differences are in bold.

## Data Availability

The data presented in this study are available on request from the corresponding author. The data are not publicly available due to privacy reason.

## Appendix A

**Table A1:** Spearman correlation coefficients between carotid IMT and laboratory data.

	ApoB	HMW Adn ^1^	LDL	Adn/ApoB Molar Ratio	Adn/LDL Molar Ratio	T-cad	Carotid IMT, Right	Carotid IMT, Left	Carotid IMT, Max.
ApoB		−0.277	0.805 *	−0.534 *	−0.468 *	−0.098	−0.058	−0.025	−0.140
HMW Adn	−0.277		−0.145	0.945 *	0.944 *	0.303	−0.075	−0.145	−0.104
LDL	0.805 *	−0.145		−0.332	−0.395 *	0.187	0.146	0.114	0.022
Adn/ApoB molar ratio	−0.534 *	0.945 *	−0.332		0.963 *	0.322	−0.046	−0.112	−0.046
Adn/LDL molar ratio	−0.468 *	0.944 *	−0.395 *	0.963 *		0.232	−0.097	−0.101	−0.058
T-cad	−0.098	0.303	0.187	0.322	0.232		0.075	0.068	−0.006
Carotid IMT, right	−0.058	−0.075	0.146	−0.046	−0.097	0.075		0.801 *	0.936 *
Carotid IMT, left	−0.025	−0.145	0.114	−0.112	−0.101	0.068	0.801 *		0.899 *
Carotid IMT, max.	−0.140	−0.104	0.022	−0.046	−0.058	−0.006	0.936 *	0.899 *	

^1^ High molecular weight adiponectin. * Significant correlations at *p* < 0.05.
